# Editorial Comment: Role of preoperative MR volumetry in patients with renal cell carcinoma for prediction of postoperative renal function after radical nephrectomy and nephron sparing surgery

**DOI:** 10.1590/S1677-5538.IBJU.2019.0217.1

**Published:** 2020-01-10

**Authors:** Luciano A. Favorito

**Affiliations:** 1 Unidade de Pesquisa Urogenital Universidade do Estado de Rio de Janeiro Rio de JaneiroRJ Brasil Professor Associado da Unidade de Pesquisa Urogenital - Universidade do Estado de Rio de Janeiro - Uerj, Rio de Janeiro, RJ, Brasil,; 2 Serviço de Urologia Hospital da Lagoa Federal Rio de JaneiroRJ Brasil Serviço de Urologia, Hospital da Lagoa Federal, Rio de Janeiro, RJ, Brasil

Lal and Collegues from India (1) in a interesting study shows the role of MRI for predicting postoperative renal function by preoperative estimation of renal parenchymal volume and correlation with glomerular filtration rate (GFR) in a prospective observational study in 30 patients with renal cancer. The MRI volumetry was used to estimate the renal parenchymal volume and shows that preoperative residual parenchymal volume on MRI renal volumetry correlates well with postoperative GFR in patients with RCC undergoing radical nephrectomy or nephron sparing surgery. Partial nephrectomy (open, laparoscopic or robotic) is considered the gold standard for treating localized renal tumors (2-6). Renal parenchymal preservation is the great objective of the partial nephrectomy. Methods that assist in programming the surgical preservation are very well coming. Recently interesting papers about the 3D printed technology shows a lot of applications in kidney surgery (7-9). Papers using the preoperative estimation of renal parenchymal volume with MRI can help a lot in planning the partial nephrectomy.
